# Similarity-Based Profiling of Hydrazone-Containing Scaffolds Active Against *Leishmania* Amastigotes

**DOI:** 10.1021/acsomega.5c05276

**Published:** 2025-10-22

**Authors:** Rita Yanka Pereira da Silva, Jheynne Laina Alves de Lima, Samara Beatriz de Abreu Pinto, Lamark Carlos I., Alessandro Kappel Jordão, Euzébio Guimarães Barbosa

**Affiliations:** Department of Pharmacy—CCS, Federal University of Rio Grande do Norte, Natal, Rio Grande do Norte 59012-570, Brazil

## Abstract

This review explores the potential of hydrazone-containing scaffolds as anti-leishmanial agents with a particular focus on their activity against intracellular *Leishmania* amastigotes. Through a strategy centered on the 3D electroshape properties of compounds, structural analysis goes beyond the traditional functional group-based approach, employing molecular alignment techniques to identify key structural features associated with anti-leishmanial activity. This review systematically compiled data from previous studies, highlighting compounds with promising in vitro activity. Structural comparisons using molecular overlays have enabled the identification of promising compounds and the exploration of their potential mechanisms of action. The integration of computational and experimental approaches provides valuable insights into the rational optimization of hydrazone scaffolds with the aim of improving efficacy, bioavailability, and safety. However, complete elucidation of the precise molecular targets and mechanisms of action remains a crucial challenge for future research. In summary, this review highlights the potential of hydrazone-containing compounds as a basis for developing novel therapeutic agents against leishmaniasis by utilizing a shape-based molecular alignment strategy to drive drug discovery efforts.

## Graphical abstract

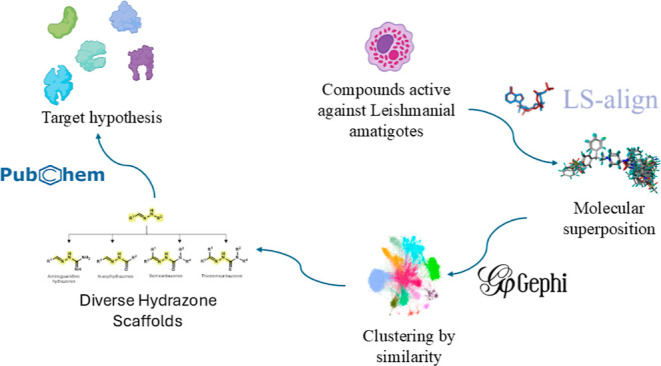

## 1 Introduction

Leishmaniasis is a parasitic disease caused by protozoa of the genus *Leishmania* and is transmitted by female phlebotomine sandflies. It manifests as four primary clinical forms: cutaneous leishmaniasis (CL), mucocutaneous leishmaniasis (MCL), visceral leishmaniasis (VL or kala azar), and postkala azar dermal leishmaniasis (PKDL). In mammalian hosts, *Leishmania* proliferates as intracellular amastigotes within phagocytic cells, particularly in macrophages. The sandflies ingest these amastigotes while feeding, which then transform into motile promastigotes in the insect gut, completing the infection cycle.^1^

According to the World Health Organization (WHO), more than one billion people are at risk of infection in leishmaniasis-endemic areas, with approximately 30 000 new cases of VL and more than one million new cases of CL occurring annually.^2^ Regions with the highest disease burden include India, Bangladesh, Nepal,^3^ East Africa, and Brazil.^4^ HIV coinfection further complicates treatment, increasing parasite burden, worsening prognosis, and elevating relapse rates.^5^

Existing therapies for VL exhibit variable efficacies and significant toxicities. Of the available treatments, only miltefosine is administered orally, while others, such as liposomal amphotericin B and paromomycin, require intravenous or intramuscular administration, posing logistical challenges in many endemic regions.^6^ There is an urgent need for simple, affordable oral therapies that are both safe and effective across different populations.^7^ In recent years, new drug combinations involving liposomal amphotericin B, paromomycin, and miltefosine have been introduced, offering improved safety and tolerability. However, these therapies remain costly, difficult to administer, and poorly stable in the high temperatures common to endemic areas.^8^ Furthermore, a disparity in efficacy persists across regions: while treatment needs are somewhat met in South Asia, drug efficacy and tolerability remain problematic in East Africa and Latin America.^9^

The ideal treatment for VL consists of a short course of oral therapy that maintains efficacy, improves tolerability, and prevents resistance. Research efforts have focused on developing combination regimens with distinct mechanisms of action to counter the emergence of drug resistance. The DNDi (Drugs for Neglected Diseases initiative) has outlined Target Product Profiles (TPPs) emphasizing the importance of therapies with high efficacy within a 10 day treatment window, effective against resistant strains, and suitable for use across various regions.^10^

The central focus of this review is on hydrazone-based compounds (Figure 1), a class of molecules recognized for their ease of synthesis and broad biological activities. These compounds include aminoguanidine hydrazones, thiosemicarbazones, *N*-acylhydrazones, and semicarbazones, which are often produced via simple condensation reactions. Despite their popularity in medicinal chemistry, only a limited number of scaffolds have advanced to clinical applications. Their structural flexibility makes them excellent candidates for optimization in anti-leishmanial drug discovery.

**Figure 1:**
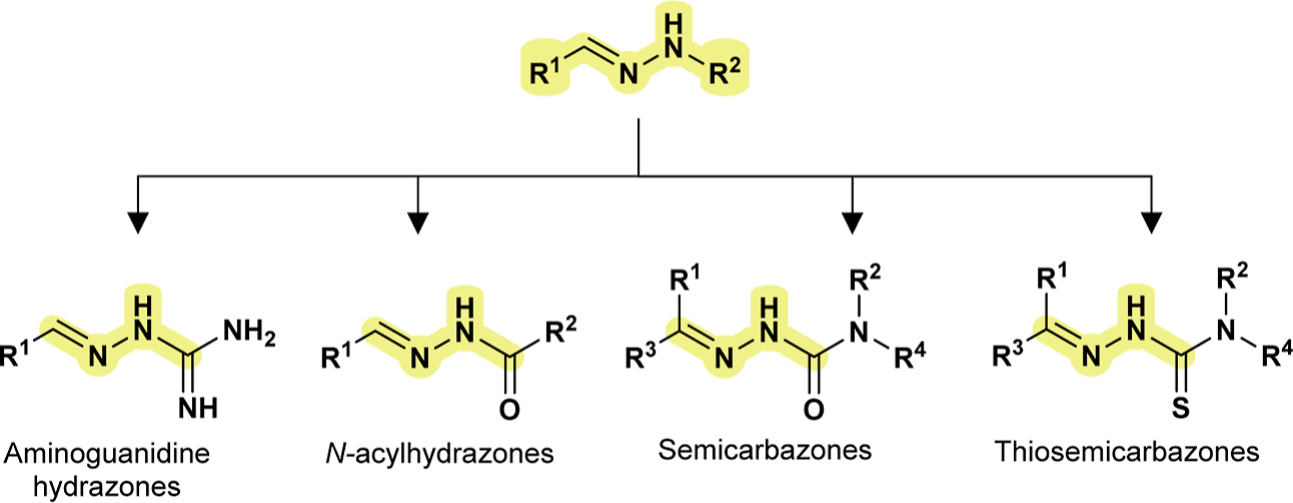
Hydrazone-based compounds.

Rather than focusing solely on molecular functional groups, this review adopts an unconventional approach by prioritizing the 3D electroshape similarities among compounds from the literature. Using molecular alignment techniques, including shape-based overlays, the review compares structurally similar hydrazone-containing scaffolds tested against amastigotes. This approach aims to uncover key insights into their potential mechanisms of action and identify promising candidates with similar properties. Furthermore, the review explores well-studied related compounds, not necessarily tested against *Leishmania*, to generate insights that may reinforce hypotheses about possible molecular targets.

This comparative reviewing approach, integrating shape-based alignments and electroshape analysis, offers a fresh perspective on reviewing the literature. By focusing not only on molecular similarity but also on local and global shape alignments, the review aims to provide researchers with new hypotheses for experimental testing. These hypotheses can be explored through in vitro assays or in silico modeling to investigate drug–target interactions and refine compound designs. The insights gained from this analysis may support the development of related compounds with improved efficacy, bioavailability, and enhanced potential as anti-leishmanial leads.

## 2 Review Strategy

This review followed a systematic strategy to compile compounds from the literature. First, the keywords “*Leishmania*,” “amastigotes,” and either “synthesis” or “natural” were used to search for relevant studies, starting from the earliest indexed paper on Scopus in 1958. The most active compounds were selected from studies using models that mimic the human phase of the parasite, including axenic amastigotes, intramacrophage amastigotes, and in vivo tests. When compounds were not close analogues, more than one representative from each study was included. Compounds were drawn based on their indicated names or directly using Marvin Sketch,^11^ and their 3D structures were optimized using the PM7 ^12^ semiempirical method in Mopac.^13^

Structural comparisons of all compounds within each series were performed using LS-align to generate molecular overlays. Proper inputs were created for the Gephi software,^14^ where nodes represented the reviewed compounds and edges indicated LS-align^15^ similarity scores. Low-similarity edges were filtered to remove unrelated nodes from the analysis. To identify direct analogues of the compounds discussed in the article, similarities were measured using the Tanimoto equation implemented in the PubChem^16^ dictionary-based binary fingerprint. These tools allowed for the correlation of the selected compounds with annotated records in the database, facilitating the identification of potential biological targets. Additionally, this approach provided common names and relevant literature for each compound, aiding in the verification of their identities and supporting assumptions regarding their biological activities.

## 3 Hydrazone-Containing Compounds and Related Scaffolds

A wide range of molecules, including all compounds from various studies, would be covered by a review focused on scaffolds containing a specific functional group tested against *Leishmania* amastigotes. However, the emphasis on 3D electroshape similarities introduces numerous possible alignments and significantly raises the computational cost of analysis. Such an extensive comparison could undermine the clarity and precision intended for this review. To ensure coherence, only one representative compound from each study was selected when others were close analogues; when structural differences were more pronounced, multiple compounds were considered. Priority was given to the most potent compound tested against amastigotes, whether axenic, intramacrophagic, or ideally in vivo, ensuring focused and meaningful analysis of the most effective molecules.

The selected compounds were grouped based on their structural similarities and visualized using Gephi^14^ to reveal connections in their molecular shapes (Figure 2). This strategy helped organize the review and illuminate meaningful patterns among the structurally related compounds. By clustering these molecules, the analysis offered the potential to form hypotheses regarding their molecular targets, providing further insights into the possible mechanisms of action. These visualized clusters also enabled a commentary on the findings reported by the original authors, offering a more comprehensive understanding of the relationships between compounds and their biological activities. This approach enhanced the clarity of the review by illustrating connections that may not have been immediately evident in individual studies. The SDF file for these compounds are available in the Supporting Information.

**Figure 2:**
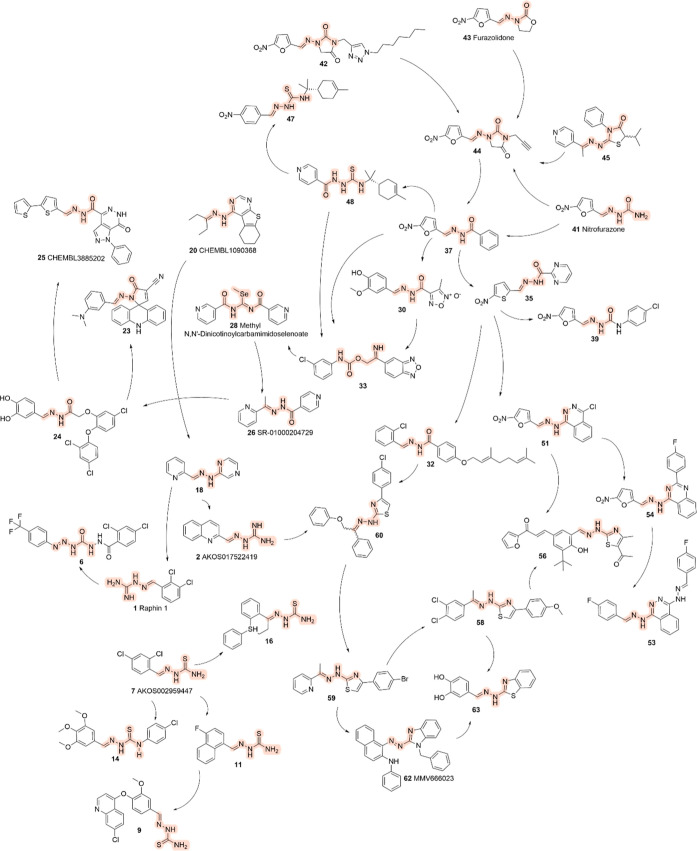
Graph presenting the structural relationship network of representative compounds from various studies, each selected for its activity against amastigote forms of *Leishmania*. The compounds are displayed with connecting edges indicating structural similarities based on three-dimensional features.

Both compounds (**1**) **Raphin 1** (Figure 3) and (**2**) AKOS017522419 (Figure 3B) were designed and evaluated for their activity against *Leishmania chagasi*.^17^ **Raphin 1** is a well-known inhibitor of the regulatory subunit PPP1R15B (R15B) of protein phosphatase, with a dissociation constant (K_d_) of 33 nM. It has previously demonstrated efficacy in a mouse model of Huntington’s disease.^18^ However, *Leishmania* lacks a direct counterpart to PPP1R15B, making it difficult to establish a direct correlation between **Raphin 1**’s mechanism of action in *Leishmania* and its known targets in other organisms. Another compound with a hydrazone scaffold that shows antiparasitic activity is (**3**) **RJ1** (Figure 3).^19^ Its activity against dihydrofolate reductase (DHFR) from *Plasmodium falciparum* seems promising, as it was able to inhibit the enzyme even in the presence of the quadruple mutant variants. Given its structural similarity to RJ1, a docking study was performed to investigate the binding behavior of Raphin 1 in the active site (Figure 4).

**Figure 3:**
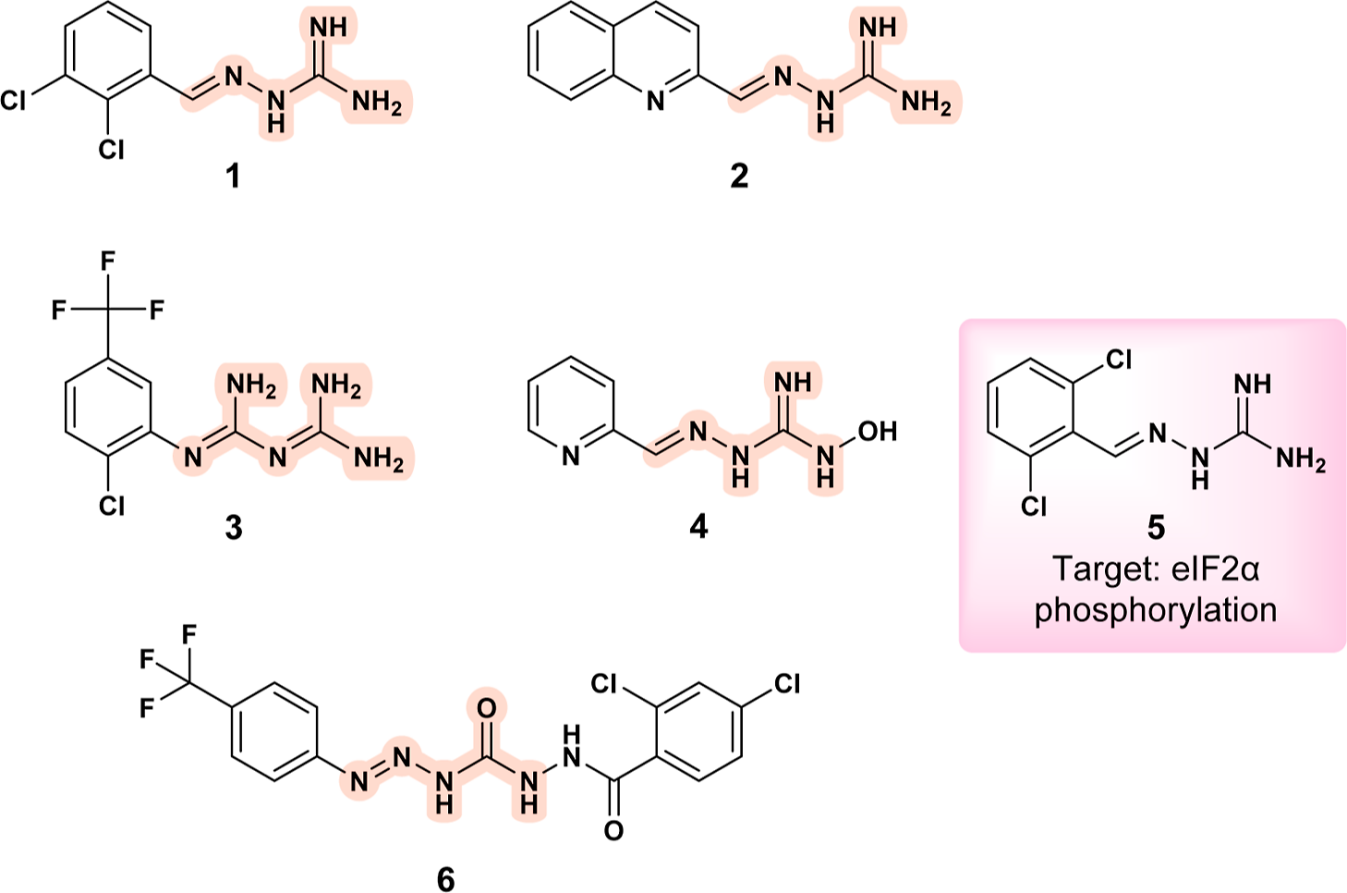
Chemical structures of selected compounds: **Raphin-1** a well-known inhibitor of the regulatory subunit PPP1R15B (R15B) of protein phosphatase (**1**); AKOS017522419 hydrazone derivative with anti-leishmanial activity (**2**); **RJ1** a structurally similar compound with antiplasmodial activity (**3**); **96826**-**57**-**2** structurally related compound (**4**); **guanabenz** a compound with potential anti-leishmanial activity through inhibition of eIF2α phosphorylation, as suggested by docking studies (**5**); carbamoyl-*N*-aryl-imine-urea derivative with reported anti-leishmanial activity (**6**).

**Figure 4:**
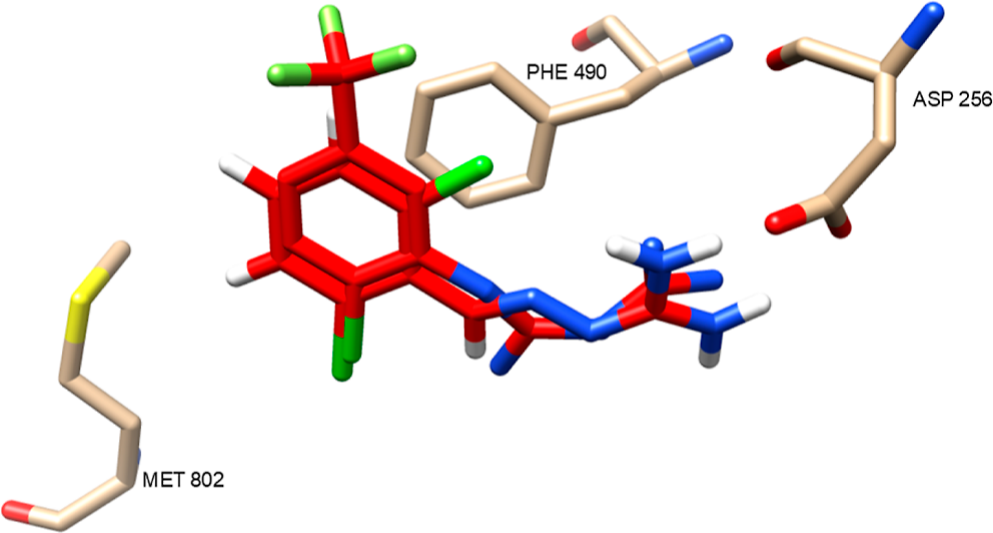
Compounds **Raphin 1** (**1**) in the active site of **RJ1** (**3**).

**AKOS017522419** ^17^ is commercially available and structurally related to **96826**-**57**-**2** (**4**) (Figure 3),^20^ which has known antiproliferative properties. These compounds share a connection to **guanabenz** (**5**) (Figure 3) with notable antiparasitic activity. **Guanabenz**, an alpha-2 adrenoceptor agonist, also showed efficacy against *P. falciparum* ^21^ and *Toxoplasma gondii* by inhibiting eIF2α phosphorylation. These compounds may act by inhibiting Leishmanial PERK, preventing eIF2a phosphorylation.^22–24^ Molecular docking performed by the authors suggested that AKOS017522419 (**2**) may interact with *Leishmania* trypanothione reductase,^17^ although this interaction has yet to be confirmed. It is plausible that, like **guanabenz**,^25^ **Raphin 1** and AKOS017522419 (**2**) may affect *Leishmania* eIF2α biochemical processes.^23^ However, further investigation is needed to verify this mechanism.

The unique **carbamoyl-*N*-aryl-imine-urea** (**6**) framework (Figure 3) was evaluated for its anti-leishmanial activity against the amastigote forms of *Leishmania amazonensis* and *Leishmania braziliensis*. In vivo tests were also conducted using a murine model of cutaneous leishmaniasis to assess the compound’s efficacy.^26^ The in vitro assays demonstrated significant leishmanicidal activity, particularly against *L. amazonensis* amastigotes. In the murine model, compound **6** showed promising results by reducing both parasite burden and lesion size, suggesting that it interferes with critical biological processes within the parasite. However, no similar compounds were identified, and a specific mechanism of action was not reported, leaving the molecular target and the precise mode of interference with parasite biology to be further investigated.

Compound **AKOS002959447** (**7**) (Figure 5) produced by the same authors^17^ was also found to be active against *Mycobacterium tuberculosis* in High-throughput tests performed by the VanderVen Lab, College of Veterinary Medicine, Cornell University (Bioassay: 1259343). Its closely related compound, **Benzalthiosemicarbazone** (**8**) (Figure 5), has demonstrated activity against *P. falciparum* dihydrofolate reductase^27^ suggesting that AKOS002959447 (**7**) might share the same target.

**Figure 5:**
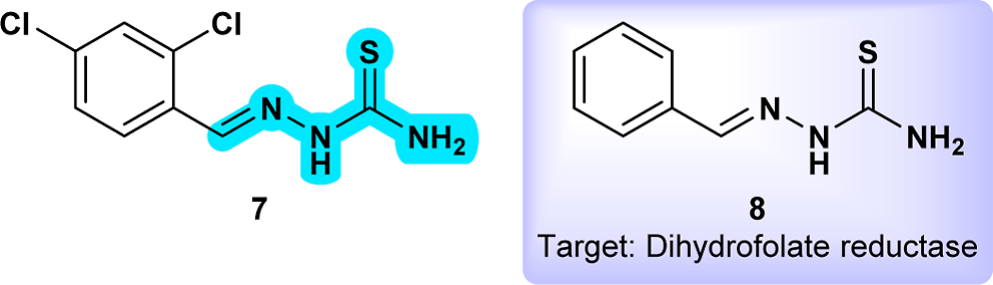
**AKOS002959447** (**7**) and related compound **Benzalthiosemicarbazone** (**8**) active against *Plasmodium*.

Compound **9** (Figure 6), a thiosemicarbazone chimerized with chloroquinoline, was tested against *Leishmania donovani* with a focus on its potential synergistic effects when combined with standard anti-leishmanial drugs miltefosine and amphotericin B.^28^ The study demonstrated that **9** exhibited significant synergy with these drugs, particularly against the amastigote form. A key finding was its ability to disrupt mitochondrial membrane potential, leading to parasite cell death, a mechanism further amplified when used in combination. The compound’s design, incorporating both chloroquinoline and thiosemicarbazone moieties, mirrors that of **CHEMBL5286667** (**10**), a structurally similar molecule with activity against *M. tuberculosis*. **10** showed low micromolar efficacy against the *M. tuberculosis* H37Rv strain and inhibited DNA gyrase.^29^ This mechanism could also apply to **9** in *Leishmania*, as topoisomerase dysfunction is linked to mitochondrial activity regulation.^30^

**Figure 6:**
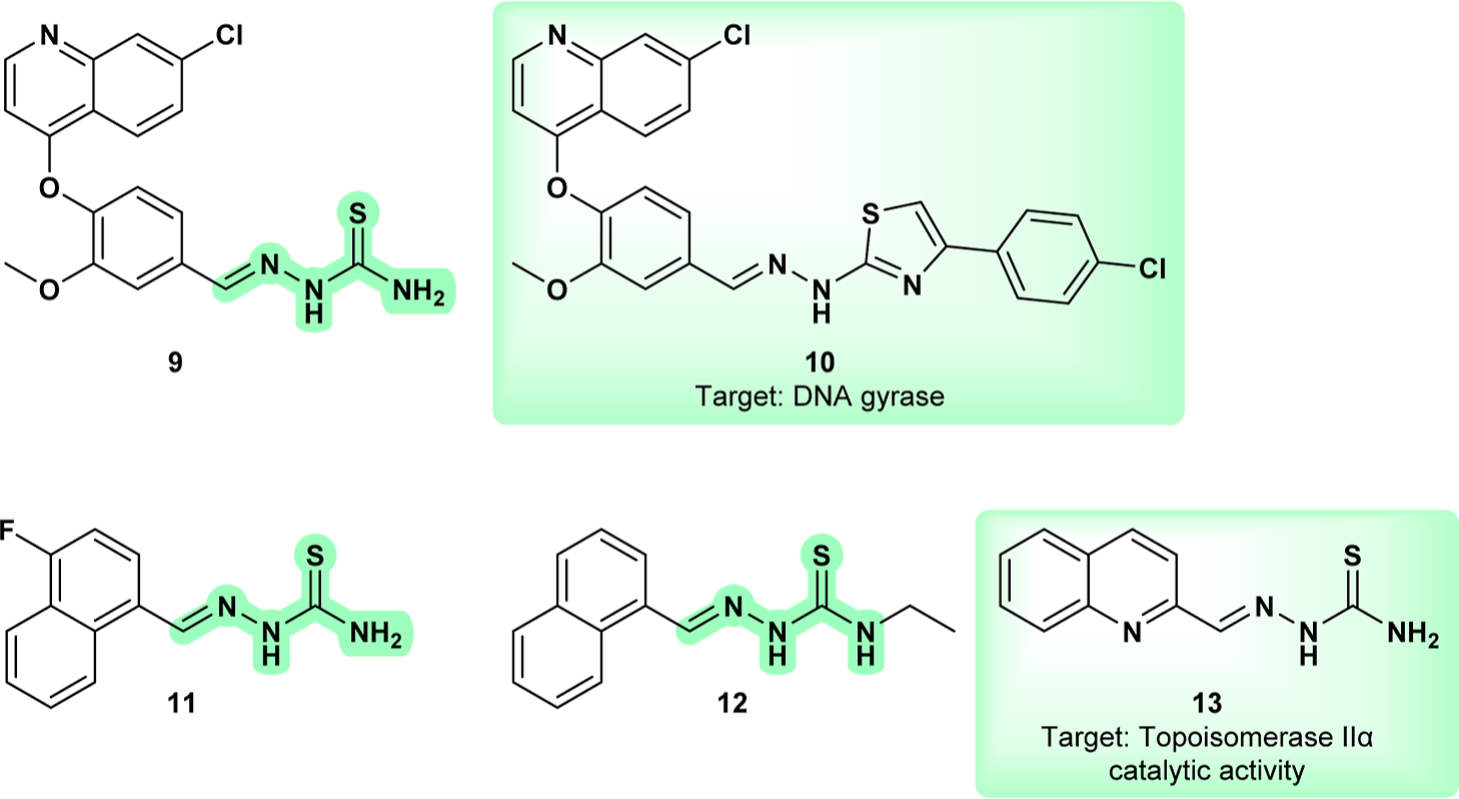
Thiosemicarbazone chimerized with chloroquinoline (**9**); **CHEMBL5286667** (**10**) DNA gyrase inhibitor; thiosemicarbazone (**11**) with high activity against *Leishmania donavani*; **CHEMBL1939440** (**12**); 2-formylquinoline thiosemicarbazone (**13**).

In another study, among 32 synthesized compounds, **11** (Figure 6) exhibited EC_50_ values below 10 μM with a selectivity index (SI) greater than 250, against *Leishmania donovani* amastigotes.^31^ **11** is closely related to the highly toxic molecule **CHEMBL1939440** (**12**) has shown acute oral toxicity and significant toxicity to aquatic life (European Chemicals Agency). The similarity to **2-Formylquinoline thiosemicarbazone** (**13**) is also notable, as this molecule has been found to disrupt DNA biochemical processes, particularly through inhibition of Topoisomerase IIα catalytic activity.^32^ These findings suggest that compounds **9** and **11** may also act on essential targets involved in the maintenance of mitotic chromosomal structure, and that **11** may exhibit similar toxicity.

In the study, **14** (Figure 7) demonstrated notable antifungal and antiparasitic activities, particularly against *Leishmania amazonensis*.^33^ **14** was more effective against promastigotes than intracellular amastigotes, likely due to the biological barriers it must overcome to reach intracellular parasites. A related analogue **CHEMBL2007612** (**15**) (Figure 7) has shown activity against tyrosyl-DNA phosphodiesterase 1 (TDP1) (Bioassay: 686978), which plays a role in mitochondrial base excision repair, an essential process for repairing oxidative damage in mitochondrial DNA. As human and *Leishmania* TDP1 share reasonable sequence identity, this enzyme could be a promising target for anti-leishmanial treatment strategies.^34^

**Figure 7:**
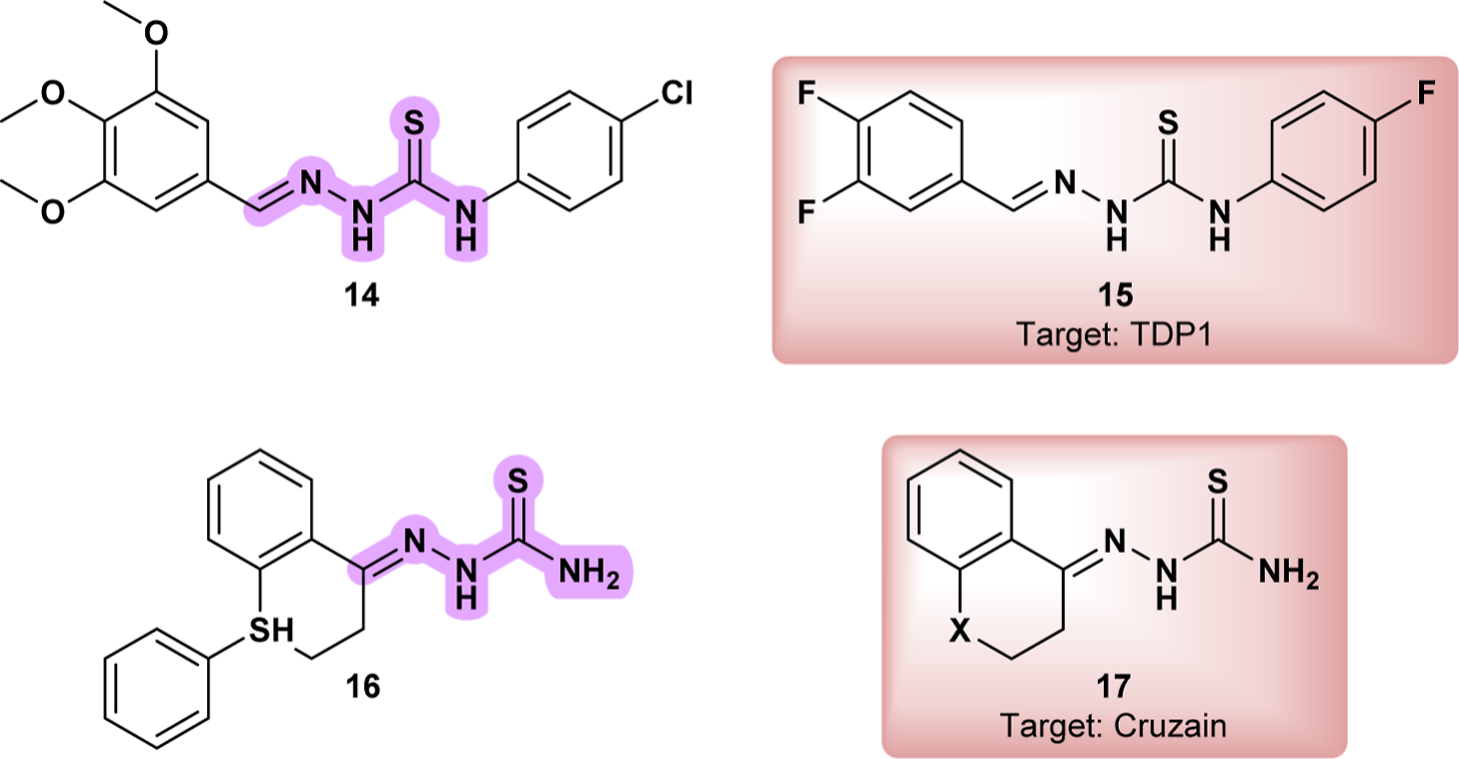
Thiosemicarbazone active against *Leishmania amazonensis* (**14**); **CHEMBL2007612** (**15**); thiosemicarbazone active against *Leishmania panamensis* amastigotes (**16**); thiosemicarbazone cysteine protease inhibitor (**17**).

Closely related hydrazone derivatives were investigated for their potential to enhance the anti-leishmanial activity of thiochroman-4-ones. The study evaluated the in vitro activity of **16** against *Leishmania panamensis* amastigotes, with a focus on identifying potent compounds with low cytotoxicity.^35^ The paper suggests that cysteine proteases, such as cathepsin L, may play a role in the mechanism of action. Similar thiosemicarbazone derivatives (**17**) were tested against *Trypanosoma cruzi* **cruzain**, aligning with the cysteine protease inhibition hypothesis.^36^

A series of related pyrazyl and pyridylhydrazone derivatives were investigated for their efficacy against *Leishmania amazonensis* and *Leishmania braziliensis* amastigotes.^37^ These compounds are structurally similar 1-[(phenylmethylidene)­amino]­guanidine,^17^ where the guanidine group is replaced the 2-pyridylhydrazone moiety, maintaining similar pharmacophoric properties. **18** (Figure 8) was found to induce reactive oxygen species (ROS) accumulation and mitochondrial membrane depolarization, leading to the disruption of energy production and apoptosis-like cell death in the parasite, without toxicity to macrophages.^37^ A closely related compound, **CHEBI:120813** (**19**) (Bioassay: 651718), has a confirmed target in *Leishmania*’s biochemical machinery—methionine sulfoxide reductase A (MsrA), an enzyme crucial for protecting the parasite against oxidative stress and supporting growth in macrophages.^38^ This aligns with authors’ findings reinforcing the role of ROS and mitochondrial dysfunction in the compound’s mechanism of action.

**Figure 8:**
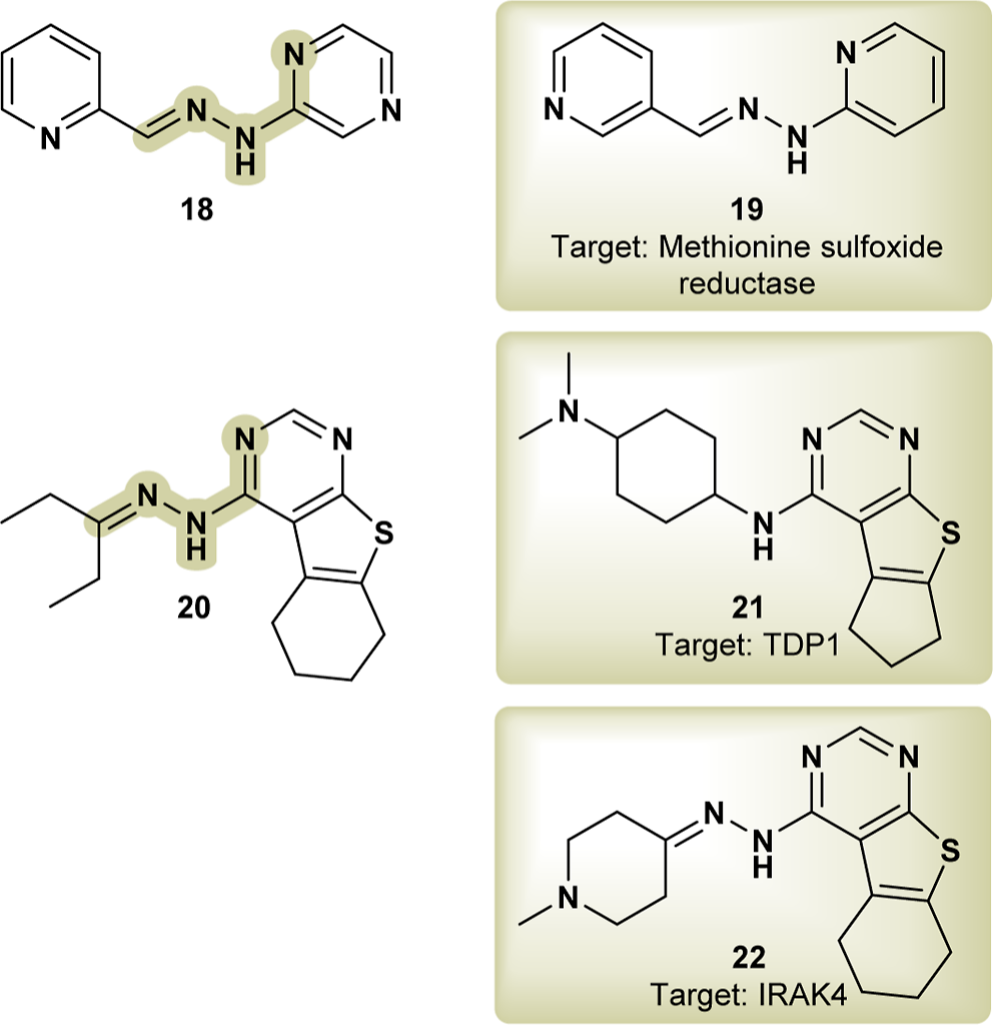
Apoptosis inducer compound (**18**); **CHEBI:120813**, MsrA inhibitor (**19**); **CHEMBL1090368** (**20**); similar to PBD ligand **6QX** (**21**); **CHEMBL1529988**, TDP1 inhibitor (**22**).

Similar tetrahydrobenzothienopyrimidine compounds, including **CHEMBL1090368** (**20**), were evaluated against *Leishmania amazonensis* ^39^ using BALB/c mice (Figure 8). However, the study did not provide detailed insights into its specific leishmanicidal mechanism of action. Compound **20** is similar to PDB ligand **6QX** (**21**),^40^ a pyrrolopyrimidine inhibitor of interleukin-1 receptor associated kinase 4 (IRAK4). IRAK4 shows 28–37% identity with a putative leishmanial protein kinase. Figure 9 shows a protein overlay comparing the active sites, and the docking simulation illustrates how compound **20** interacts with its binding site. Interestingly, **CHEMBL1529988** (**22**) (Figure 8), a closely related compound, was found to inhibit tyrosyl-DNA phosphodiesterase 1 (TDP1), a target also implicated in the action of **15** (Figure 7). TDP1 may be a potential shared molecular target for these compounds.

**Figure 9:**
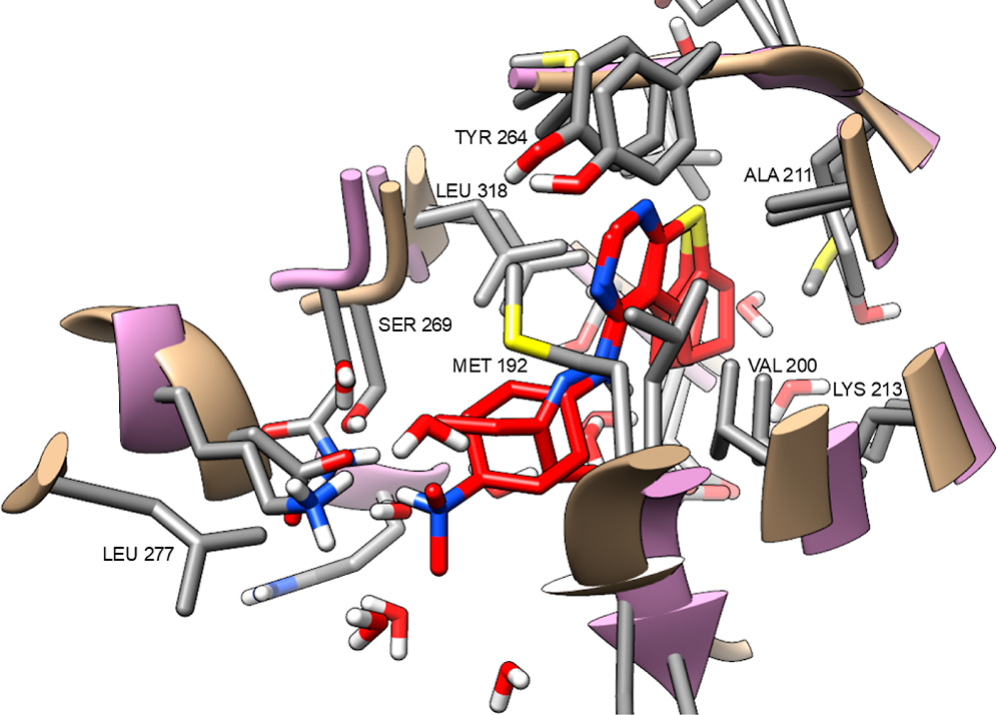
Compound **20** in the active sites of IRAK4 in the presence of the ligand **6QX** (**21**).

Compounds **23**,^41^ **24**,^42^ and **CHEMBL3885202** (**25**)^43^ (Figure 10) are unique, as no structurally similar scaffolds have been tested to draw definitive conclusions about their biological targets in *Leishmania*. The authors hypothesized several biological targets for the spiro-acridine derivative **23**, including trypanothione reductase (TryR), *Leishmania donovani* topoisomerase I (LdTopoI), and CYP51; however, no confirmatory tests were conducted. The precise mechanism of action for compound **24** remains unconfirmed. Additionally, **25** has been reported to induce increased ROS production, cell shrinkage, phosphatidylserine exposure, and DNA fragmentation, hallmark indicators of apoptosis-like cell death in the parasite.

**Figure 10:**
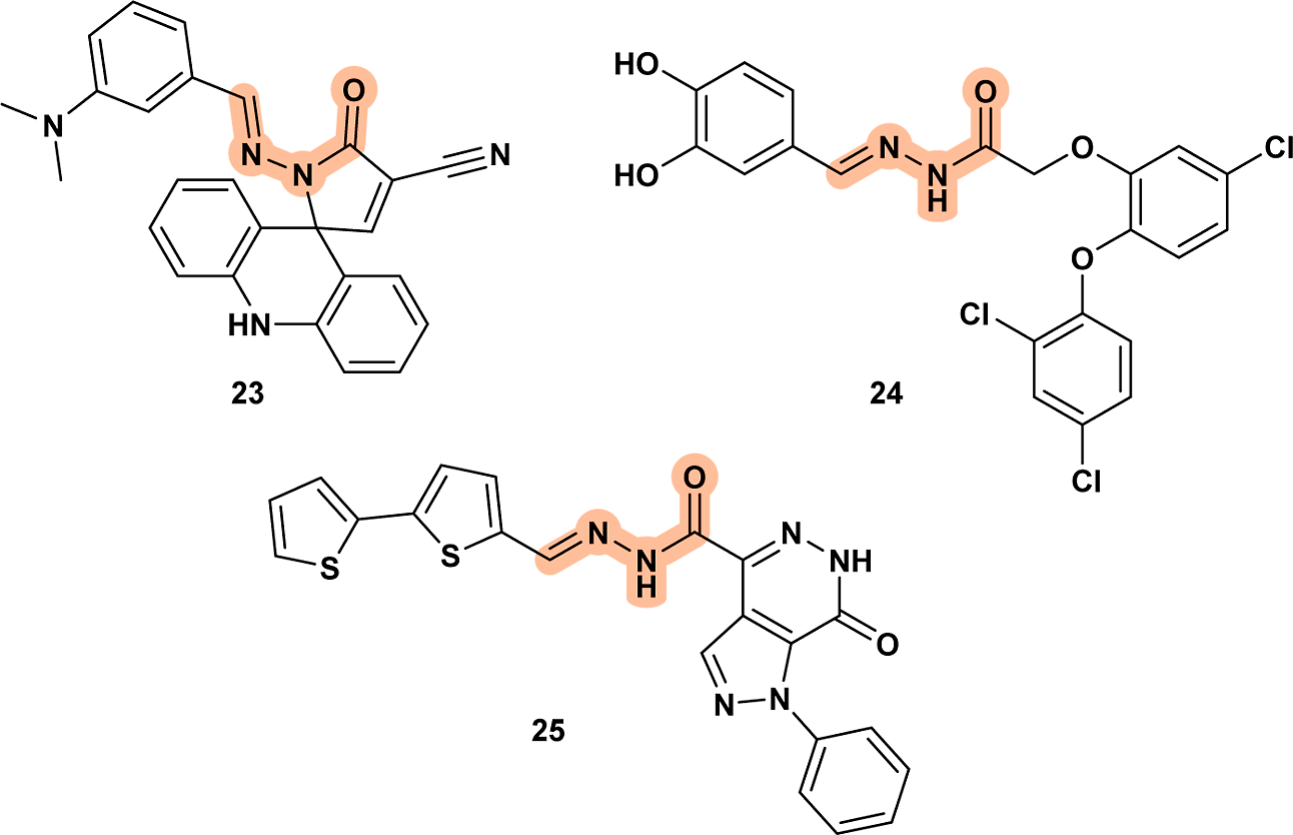
Compounds **23**, **24** and **25**.

The cytotoxicity and anti-leishmanial activity of isoniazid-derived hydrazones and 2-pyrazineformamide thiosemicarbazones against *Leishmania braziliensis*, identifying **SR-01000204729** (**26**) (Figure 11) as having a favorable balance between cytotoxicity and anti-leishmanial activity.^44^ **PCIH** (**27**) (Figure 11), which is nearly identical to **26**, is a tridentate chelator used in managing iron-overload diseases.^45^ This iron interaction may be relevant in explaining **26**’s anti-leishmanial effect. Iron plays a critical role in host–pathogen interactions, as intracellular pathogens like *Leishmania* rely on host Fe for survival, growth, and virulence. *Leishmania* disrupts iron sequestration into ferritin by cleaving Fe-chaperones such as poly­(rC)-binding proteins,^46^ thereby promoting intracellular growth. It can be hypothesized that **26** may interfere with these processes, potentially inhibiting the parasite’s ability to utilize host iron. However, further studies are required to confirm this proposed mechanism, though it is supported by existing literature.

**Figure 11:**
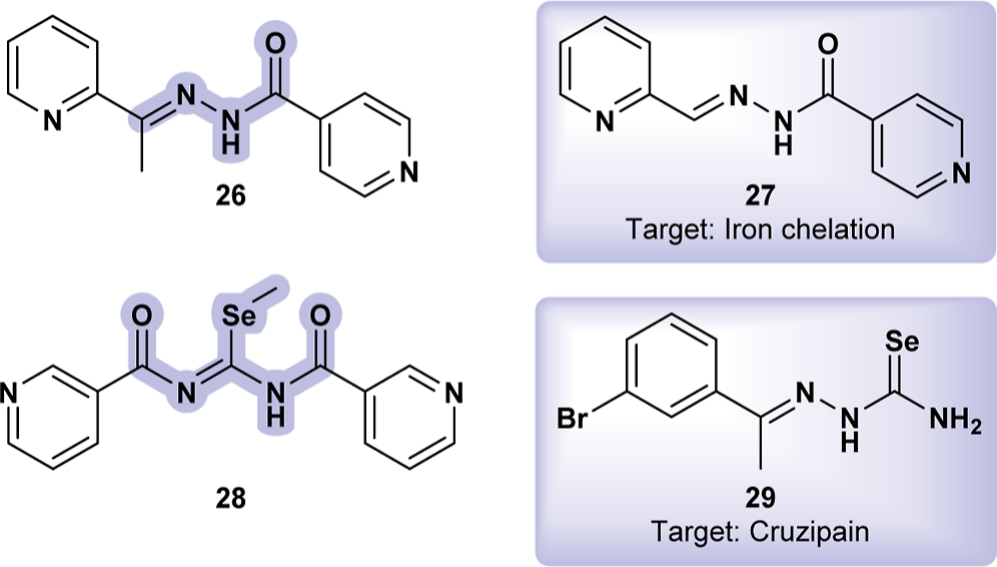
**SR-01000204729** (**26**) and its close compound PCIH (**27**); **Methyl-*N*,*N*′-dinicotinoylcarbamimidoselenoate** (**28**); active compound against Cruzipain (**29**).

**Methyl-*N*,*N*′-dinicotinoylcarbamimidoselenoate** (**28**) (Figure 11), a related imidoselenocarbamate, was investigated for its anti-leishmanial potential against *Leishmania infantum*.^47^ **28** demonstrated moderate anti-leishmanial activity with low toxicity to host cells, making it a promising candidate for further development. Selenosemicarbazones and similar compounds have been extensively reviewed^48^ highlighting their predominant exploration for antichagasic activity. For instance, a selenosemicarbazone (**29**) showed high potency against Cruzipain, a crucial cysteine protease in *T. cruzi*.^49^ Given the structural similarity, cysteine peptidase A (CPA, XP_001465113.1) in *L. infantum* may serve as a closely related target, suggesting that selenosemicarbazones could potentially be their inhibitors.

Among a series of hybrid furoxanyl *N*-acylhydrazone derivatives as potential drug candidates against *Leishmania amazonensis*, one of them, compound **30** (Figure 12), demonstrated superior selectivity and greater potency, with a selectivity 50-fold higher than that of Amphotericin B.^50^ Its structure is closely related to oxadiazole **CHEMBL3196729** (**31**) (Figure 12), which is active against *Leishmania mexicana* pyruvate kinase (Bioassay: 1721), further supporting the potential of compound **30** to exhibit similar properties.

**Figure 12:**
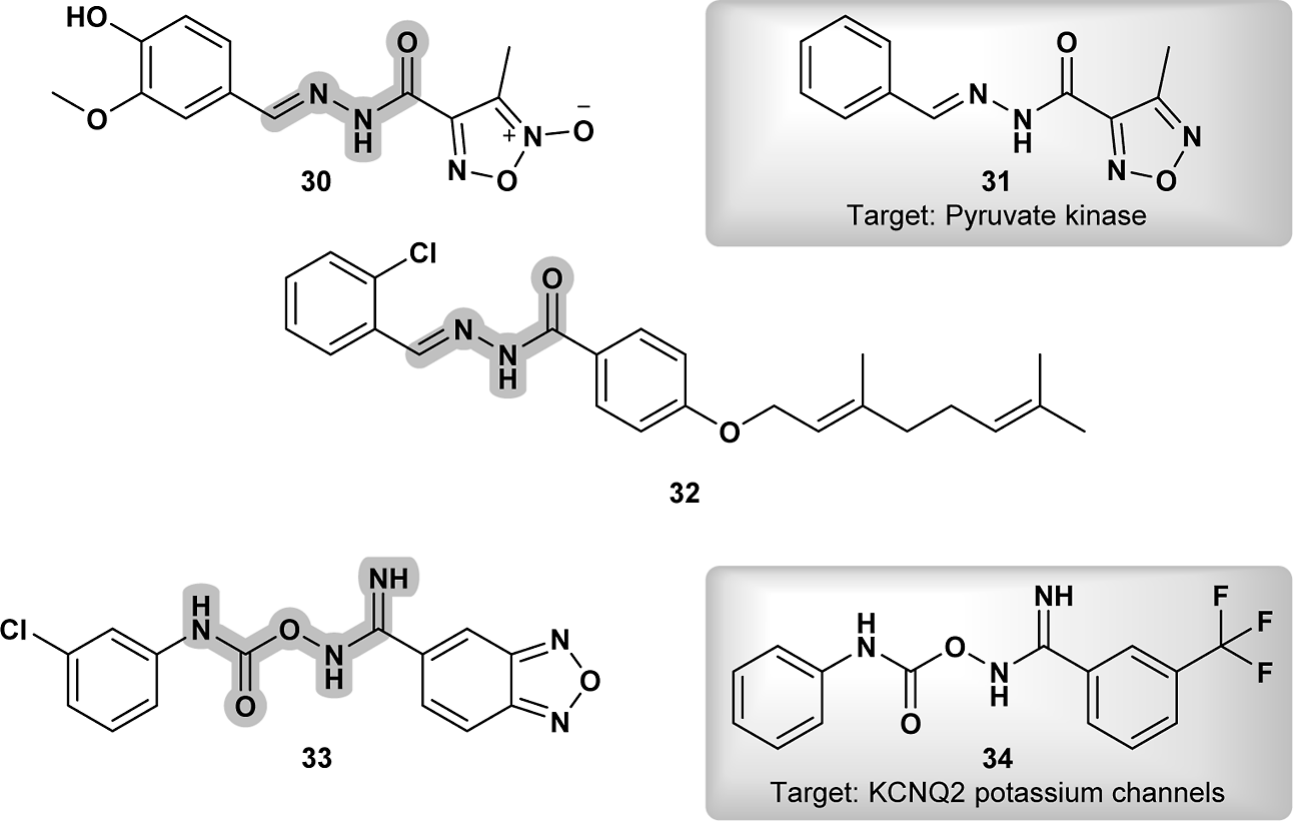
High selective compound against *Leishmania amazonensis* (**30**) and its closely structure **CHEMBL3196729** (**31**); compound with extensive antiprotozoal efficacy (**32**); 33 and its closely related compound **MLS001146594** (**34**) active against KCNQ2 potassium channels.

The unique compound **32** (Figure 12) was extensively evaluated for its antiprotozoal efficacy, particularly through in vitro assays against various parasitic protozoans, including *Trypanosoma brucei*, *T. brucei rhodesiense*, *T. cruzi*, and *Leishmania infantum*.^51^ The compound demonstrated significant activity against *L. infantum*, positioning it as a promising candidate for anti-leishmanial therapy. Its structural features, especially the presence of a chlorine substituent, were noted as potential contributors to its broad-spectrum activity, likely by disrupting essential metabolic or signaling pathways required for parasite survival. Notably, no closely related compounds were found in the PubChem database, suggesting the compound’s novelty and potential for further exploration.

An interesting related scaffold, carboxyimidamide-substituted benzo­[*c*]­[1,2,5]­oxadiazoles, demonstrated promising activity, with compound **33** (Figure 12) proving to be active against *Leishmania donovani*.^52^ A structurally related carboxyimidamide **MLS001146594** (**34**) (Figure 12) is well-known for its action on KCNQ2 potassium channels, which are also present in *L. donovani* (CAJ1987321.1). These putative potassium channels may be involved in several cellular pathways,^53^ suggesting a potential mechanism of action for **33** (Figure 12).

Compound **35** (Figure 13), derived from a series of 2-pyrimidinyl hydrazones, was evaluated for its activity against *Leishmania amazonensis*, with a focus on mitochondrial dysfunction and ROS production as part of its mechanism of action.^54^ The study indicated that the mitochondrial membrane is a critical target, as the compound induces mitochondrial depolarization and disrupts the membrane potential. Additionally, ROS production increased following treatment, further contributing to parasite death. A closely related compound, **CHEMBL1974036** (**36**) (Figure 13), was extensively tested (Bioassay: 624296), although no conclusive information on its mechanism of action was reported.

**Figure 13:**
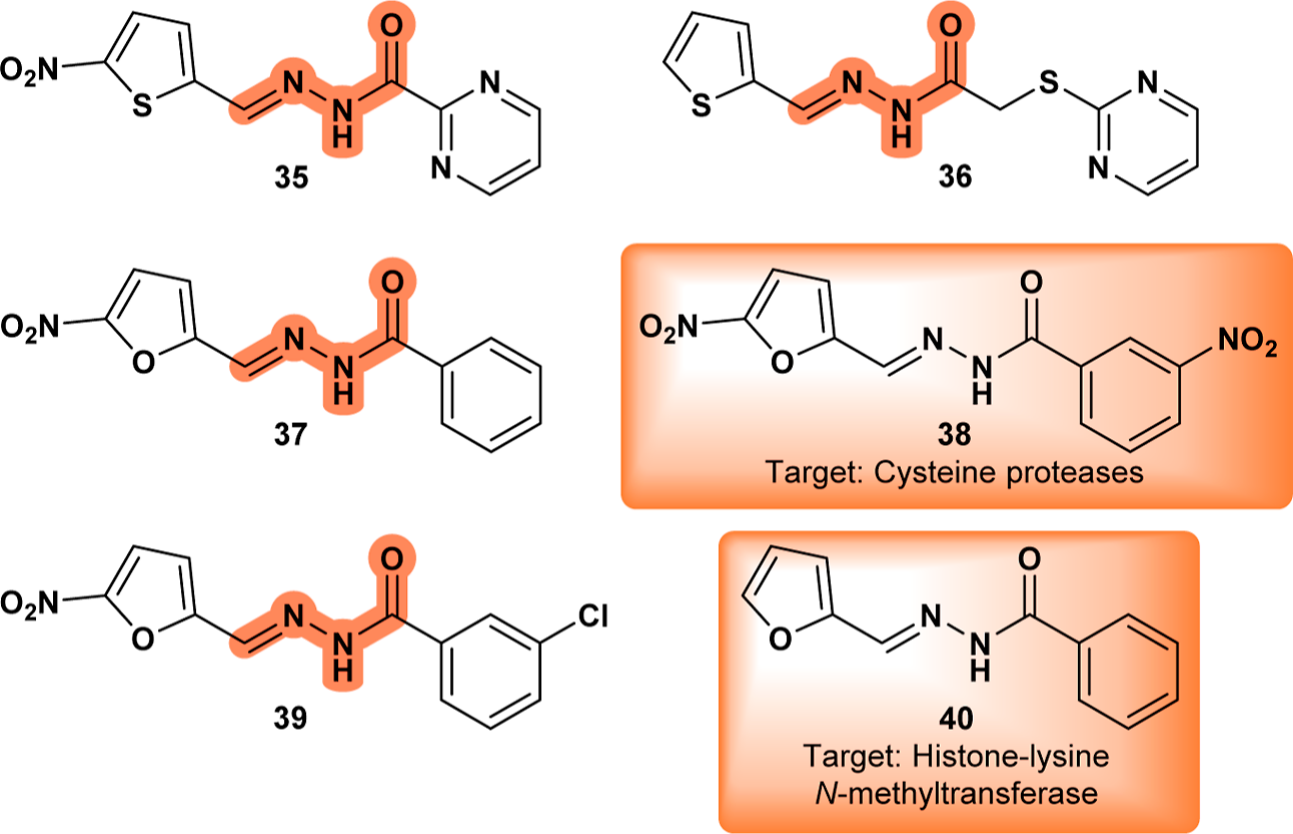
2-Pyrimidinyl hydrazone derivative (**35**); **CHEMBL1974036** (**36**); semicarbazone derivative (**37**); **CHEMBL416237** (**38**); semicarbazone derivative (**39**); **CHEMBL3198318** (**40**).

Compound **37** (Figure 13) has an established scaffold with known antiparasitic properties against *Leishmania* promastigotes and amastigotes, continuing a long tradition of research into related structures.^55^ **CHEMBL416237** (**38**), for instance, was found to have antimalarial and antichagasic activity by inhibiting cysteine proteases.^56^ **37** showed IC_50_ values lower than those of the reference drugs pentamidine and amphotericin B. However, unlike **38**, the study proposed that the primary mechanism of action was the reduction of the nitro group by unspecific nitroreductases, without specifically implicating cysteine protease targets.

A related semicarbazone derivative **39** (Figure 13) was tested in vitro and in vivo against *Leishmania amazonensis* and *Leishmania braziliensis* amastigotes. It exhibited potent leishmanicidal activity, significantly reducing lesion size in BALB/c mice with intraperitoneal administration.^57^ The authors suggested that the mechanism of action involves mitochondrial dysfunction, induction of apoptosis through caspase-like activity, and autophagy. The compound was reported to cause mitochondrial membrane depolarization and activate apoptosis-like pathways in Leishmania. A conjectural molecular target may involve a mechanism similar to that of **CHEMBL3198318** (**40**) (Figure 13), which was tested against human euchromatic histone-lysine *N*-methyltransferase 2 (Bioassay: 504332) and has a counterpart in Leishmania, the **ankyrin repeat protein** (XP_003723231.1).

**Nitrofurazone** (**41**) (Figure 14) is an older compound known for its significant activity against amastigotes.^58^ **41** likely exerts its antileishmanial activity through a mechanism similar to its action in bacteria, undergoing enzymatic reduction, potentially mediated by a leishmanial reductase. This reduction process converts nitrofurazone into reactive intermediates that can covalently bind to proteins and possibly nucleic acids, leading to damage to essential cellular macromolecules. The reduction process relies on the presence of NADPH or NADH to facilitate nitrofurazone’s activation.^59^ Inspired by nitrofurazone, compounds **42** and **44** (Figure 14) were evaluated for their in vitro antileishmanial activity against *Leishmania donovani* and *Leishmania major*, targeting both promastigote and amastigote forms. Compounds **42** and **44** exhibited significantly better activity against *Leishmania donovani* amastigotes compared to the parent drug, **nitrofurazone**, demonstrating success in optimizing antileishmanial drug design. These findings highlight their potential as promising candidates for further development in the treatment of leishmaniasis.^60^

**Figure 14:**
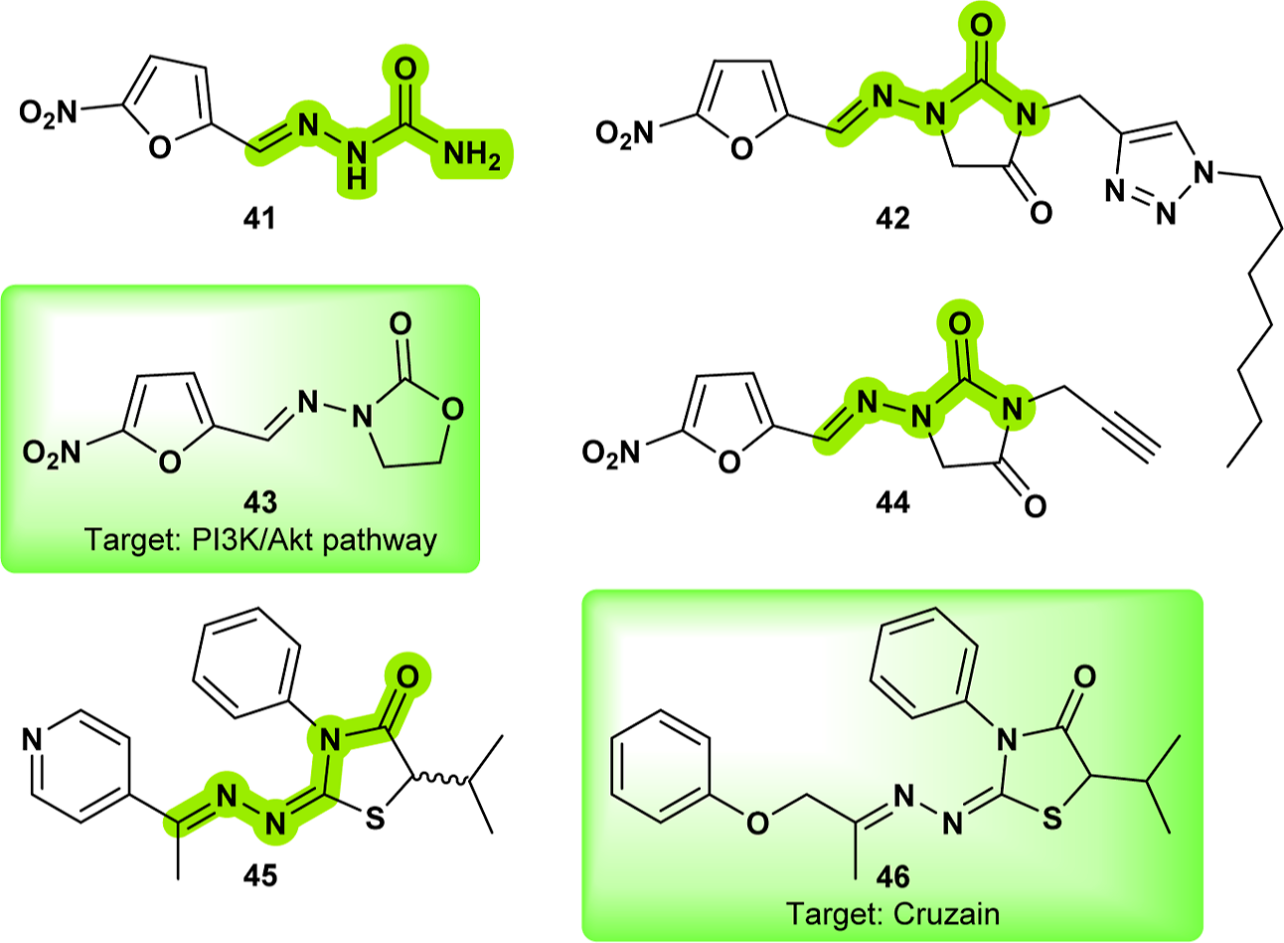
**Nitrofurazone** (**41**); optimized structures from nitrofurazone (**42**), (**44**); PI3K/Akt pathway suppressor **furazolidone** (**43**) and its electroshape-related compound (**45**), cruzain inhibitor **CHEMBL2180342** (**46**).

**Furazolidone** (**43**) (Figure 14), a classical antibacterial drug,^61^ was evaluated for antileishmanial activity against several *Leishmania* species, focusing particularly on *Leishmania chagasi*. Compound **43** displayed potent activity against *L. chagasi* intracellular amastigotes, although it exhibited cytotoxicity at higher concentrations. The drug induced mitochondrial swelling, vacuolization, and nuclear damage, leading to the loss of intracellular organelles and parasite death.^62^ This activity is attributed to its ability to induce apoptosis through a reactive oxygen species (ROS)-dependent mitochondrial signaling pathway and suppression of the PI3K/Akt pathway.^63^ While this mechanism can result in undesirable cytotoxic effects, the development of safer analogues could improve its therapeutic profile, making it a promising lead for new antiparasitic drug development. Compound **43** was identified as having low electroshape similarity to compound **45** ^64^ that displayed promising activity against *L. amazonensis* amastigotes. Compound **45** shared structural similarity with **CHEMBL2180342** (**46**), which exhibited significant activity against *T. cruzi* through cruzain inhibition.^65^ This similarity suggests that the inhibition of cysteine proteases in *L. amazonensis* may similarly contribute to the observed activity.

Compound **47** (Figure 15), a 4-nitrobenzaldehyde thiosemicarbazone derived from *S*-limonene, was evaluated against *Leishmania amazonensis*. It exhibited greater toxicity toward the parasite than toward mammalian J774A1 macrophages, inducing significant ultrastructural changes, including mitochondrial swelling, disorganization of the inner mitochondrial membrane, accumulation of lipid bodies, and cytoplasmic vacuolization.^66^ The same research group also investigated a related limonene-acylthiosemicarbazide hybrid, compound **48** (Figure 15), which demonstrated the most potent antiproliferative activity and a higher selectivity index for intracellular amastigotes than **47**. **48** primarily targeted the Golgi complex of the parasite, causing structural disorganization and vesiculation in the flagellar pocket. These ultrastructural changes suggest that the compound may interfere with lipid biosynthesis and secretion pathways.^67^ Compound **CHEMBL3235021** (**49**) (Figure 15), a structurally related thiosemicarbazone, exhibited excellent anticancer properties, making it one of the most relevant analogues in the literature.^68^ Numerous antiproliferative properties are attributed to the thiosemicarbazone class and are listed on PubChem. Uncovering the precise mechanism of action of these derivatives could pave the way for the development of potent antiparasitic drugs as well.

**Figure 15:**
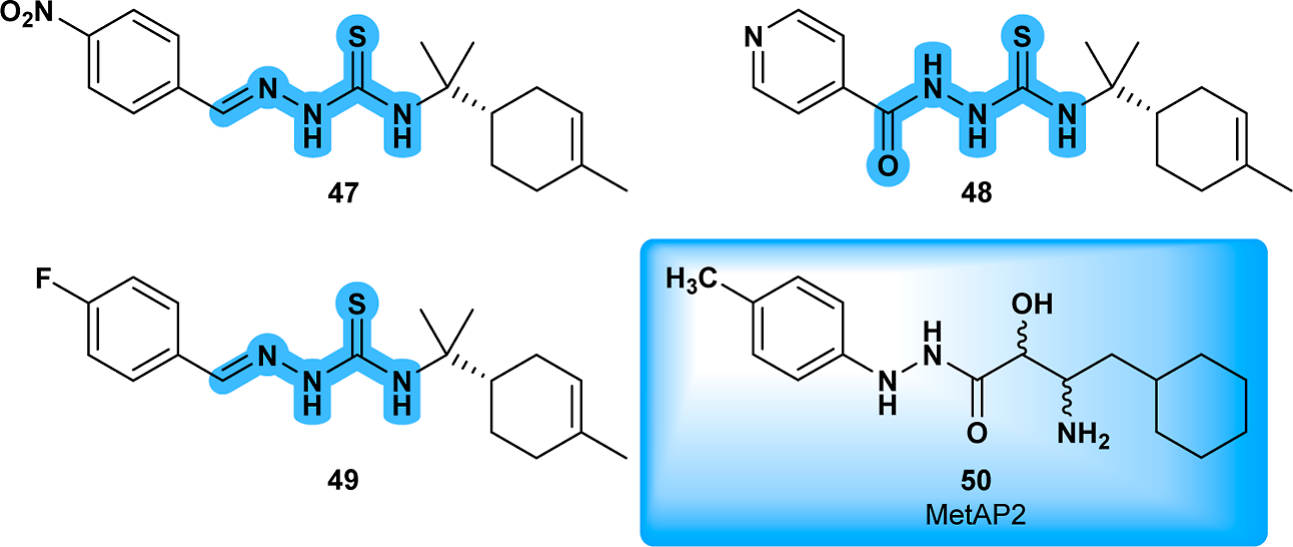
**47** and **48** and their structure related-compound **49**; **AO2** (**50**), another closely related-compound and MetAP2 ligand.

A docking study was performed to investigate the interaction of compound **46** in the active site of methionine aminopeptidase 2 (**A0A3S7WWI2**), using the structurally similar ligand **AO2** (**50**) as a reference (Figure 16).

**Figure 16:**
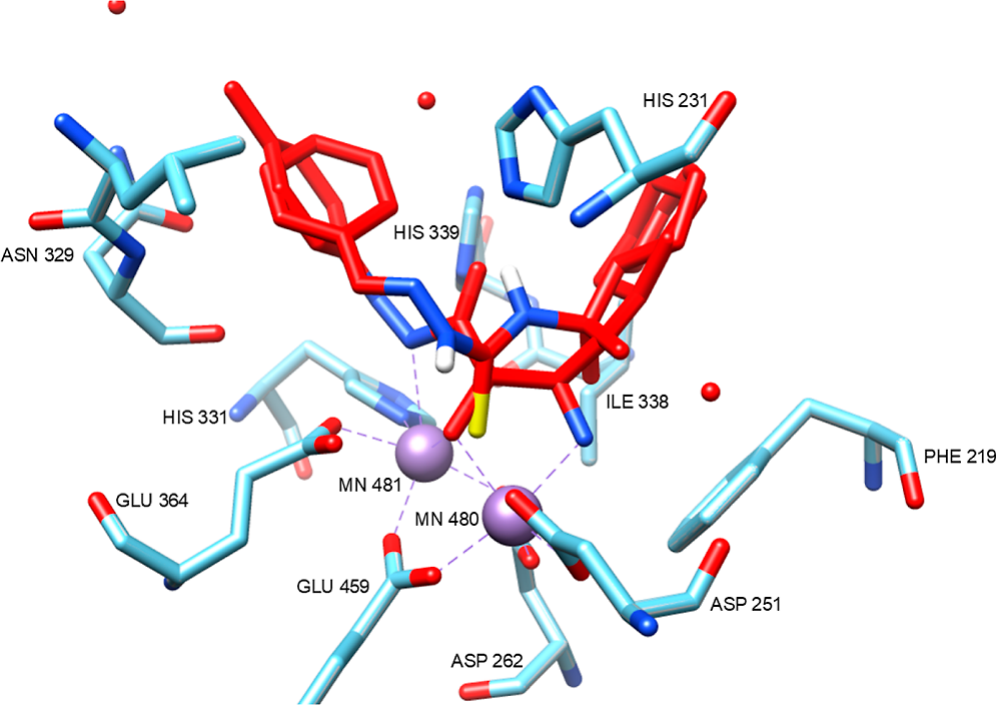
Compound **46** docked in the MetAP2 active site.

Compound **51** (Figure 17), a phthalazine derivative and closed-ring *N*′-(furan-2-ylmethylideneamino) benzenecarboximidamide, was tested in vitro for its antileishmanial activity against *Leishmania braziliensis*.^69^ This compound, a closed-ring structure analogue similar to compounds **18** (Figure 8)^37^ and **49** (Figure 15),^39^ is thought to exert its effects through oxidative stress and mitochondrial dysfunction. It induces oxidative stress in the parasite, leading to impaired mitochondrial dehydrogenase activity. Although superoxide dismutase (SOD) was initially proposed as a target, molecular docking studies revealed weak interactions with this enzyme, suggesting that the compound may act through alternative mechanisms.^69^

**Figure 17:**
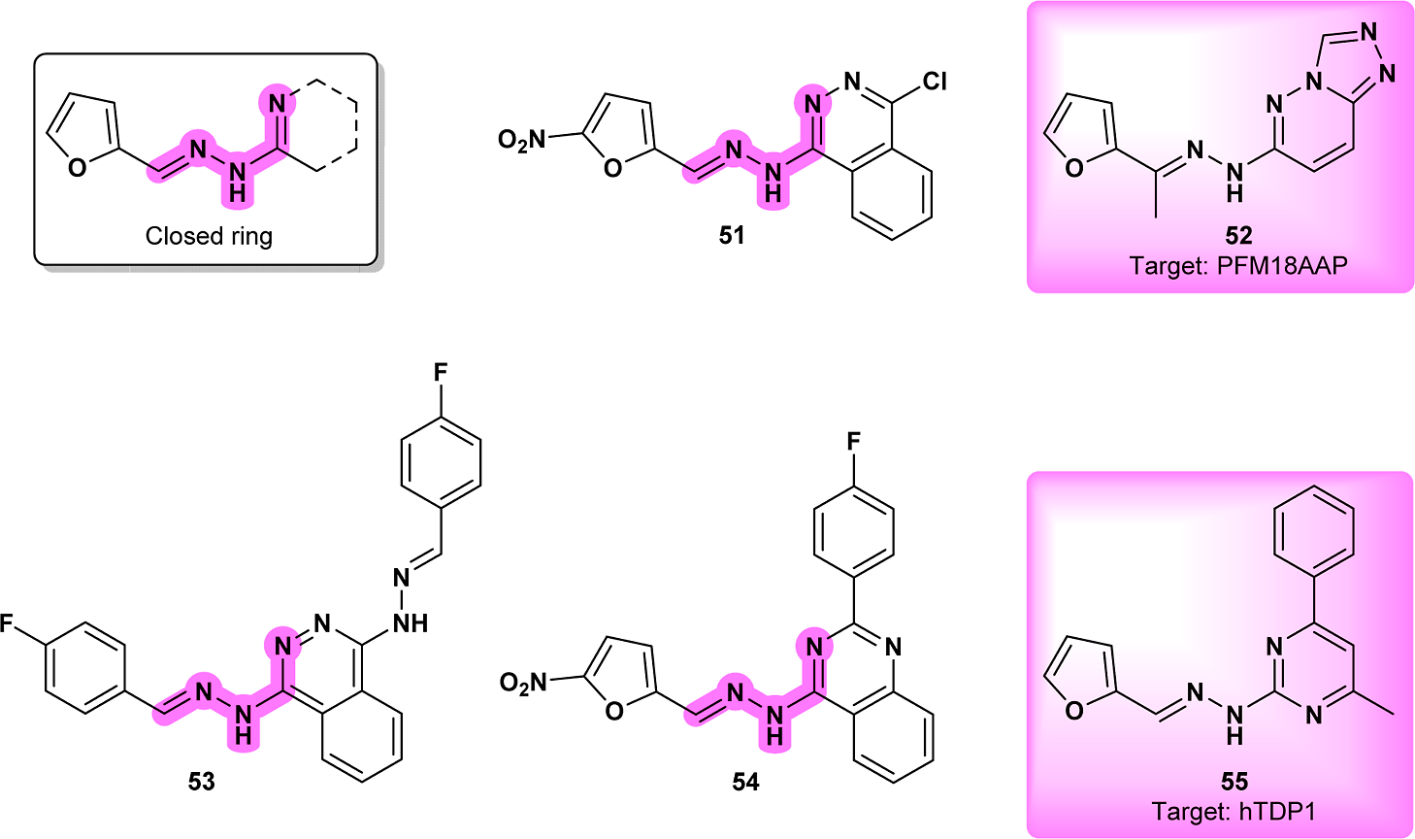
PFM18AAP inhibitor (**52**) structure related to **53** and **51**; (**55**) and its analogue (**54**) hTDP1 inhibitor.

Compound **53** (Figure 17), a 1,4-bis­(substituted benzalhydrazino)­phthalazine derivative, was tested against *Leishmania braziliensis* and *Leishmania mexicana*. It was evaluated using murine macrophages, and molecular docking studies were conducted to investigate potential interactions with superoxide dismutase (SOD).^70^ **53** showed promising results, exhibiting better antileishmanial activity against *L. braziliensis* compared to the reference drug **Glucantime**. However, enzymatic inhibition assays for SOD demonstrated poor inhibitory activity for the active compounds, confirming that SOD inhibition is not the mechanism of action. **CHEMBL3212988** (**52**) (Figure 17), a structurally similar compound to **51** and **53**, is known to inhibit *P. falciparum* M18 Aspartyl Aminopeptidase (PFM18AAP). However, a homologous target in *Leishmania* (XP_001566576.1) shows low sequence identity, making it a less likely candidate for activity in *Leishmania*, and thus a distant possibility.^71^

Compound **54** (Figure 17) was proposed to target folate pathways in *Leishmania*, with mechanistic assays showing interactions with pteridine reductase 1 (PTR1) and dihydrofolate reductase-thymidylate synthase (DHFR-TS) in *Leishmania infantum*.^72^ A closely related analogue, **CHEMBL3195759** (**55**) (Figure 17), demonstrated similar activity, particularly against TDP1 in human cells.^73^ TDP1 repeatedly emerges as a promising target for the reviewed compounds due to its critical role in repairing oxidative damage in mitochondrial DNA.^74^

Benzylidene-hydrazineyl-thiazole **56** (Figure 18) can be considered part of the same closed-ring analogue, now incorporating a bioisosteric thiazole ring. This compound induced reactive oxygen species (ROS) and nitric oxide (NO) production, which led to apoptosis-like death in *Leishmania* amastigotes. The compounds triggered the externalization of phosphatidylserine, a hallmark of apoptosis, in treated parasites. In vivo, compound **56** demonstrated a 73% reduction in parasite load in the spleens of infected hamsters.^75^ It shares remarkable structural similarities with the antifilarial chalcone-thiazole derivative **CHEMBL3260651** (**57**), which exhibited promising activity against *Brugia malayi*, demonstrating 100% embryostatic effects and moderate microfilaricidal activity in in vivo models.^76^ However, the precise mechanism of action for **57** remains unknown. Further research is needed to clarify the exact mechanisms by which these compounds exert their antiparasitic activities.

**Figure 18:**
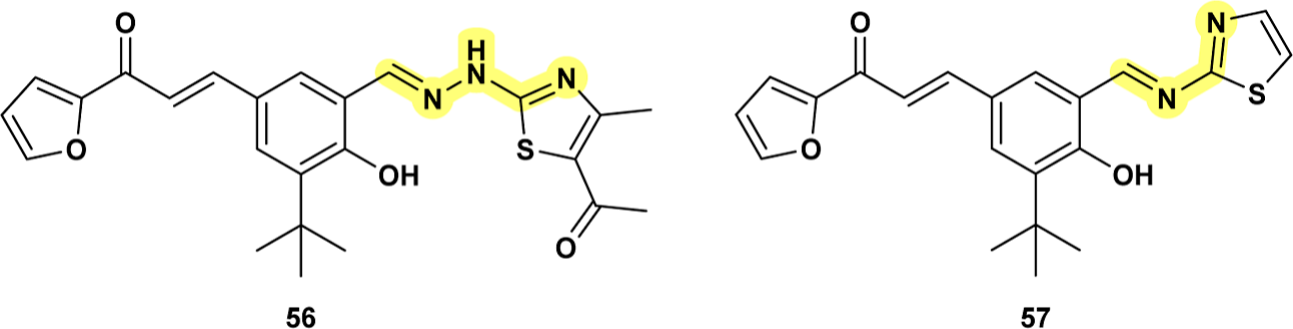
Compound **56** and its analogue **CHEMBL3260651** (**57**).

Structurally related compounds **58** ^77^ and **59** ^78^ (Figure 19) demonstrated potent activity against both promastigotes and amastigotes of *Leishmania infantum*. These compounds caused significant morphological changes in the parasites, including mitochondrial swelling, cellular disorganization, and direct structural damage, nearly eliminating macrophage infection at the highest concentration tested (1 mg/mL). **60** (Figure 19), a branched analogue of **58** and **59**, also showed strong antileishmanial effects against *L. major* promastigotes and amastigotes, with potency approximately six times greater than the standard drug **Glucantime**.^79^ These compounds share structural similarities with **CHEMBL3194563** (**61**) (Figure 19), which has been tested across multiple biological assays, showing activity against malarial parasites and *T. cruzi* replication. TDP1 emerged again as a possible target for these compounds.^73^

**Figure 19:**
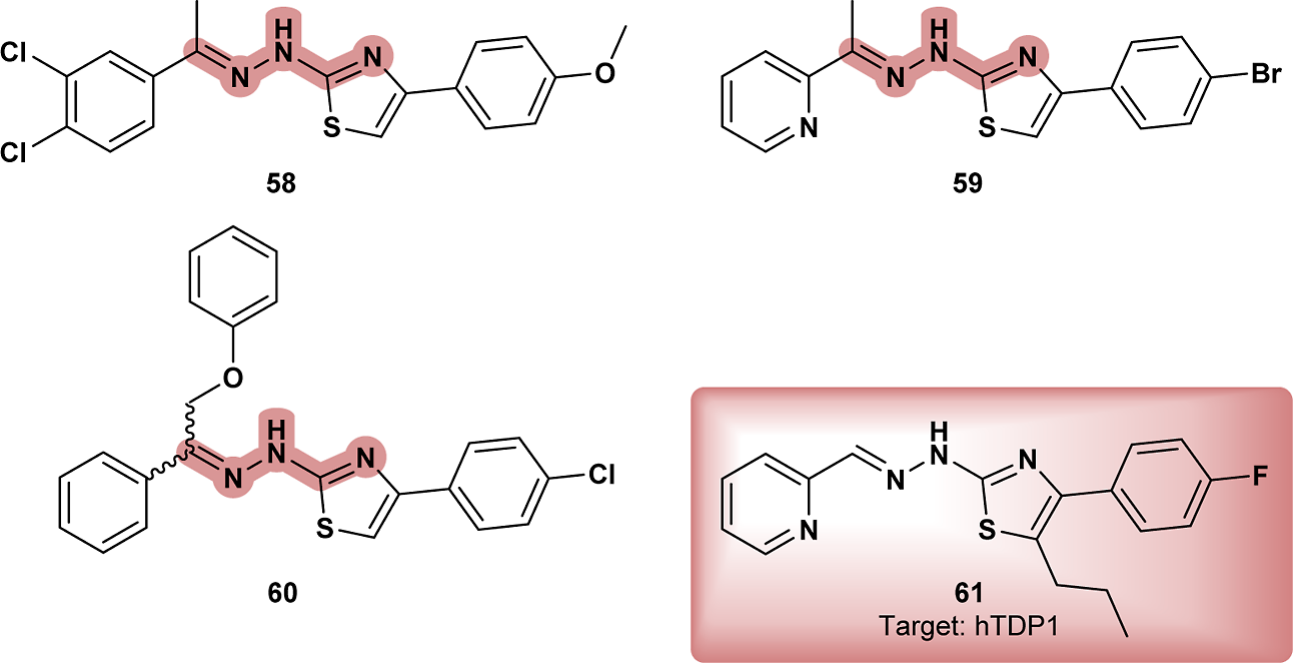
**58**, **59** and their branched analogue (**60**); **CHEMBL3194563** (**61**).

In a study investigating the antileishmanial activity of compounds from the Medicines for Malaria Venture (MMV) Malaria Box collection against intracellular *Leishmania major* amastigotes, compound MMV666023 (**62**) (Figure 20) emerged as active in an intracellular assay using luciferase-expressing parasites to measure proliferation.^80^ As with typical screening protocols, no specific molecular targets were investigated at this stage, leaving the mechanisms of action for **62** unexamined. However, in another study, **62** was evaluated for its potential to inhibit *P. falciparum* deoxyhypusine hydroxylase (DOHH), an enzyme critical for the biosynthesis of hypusine, a modification essential for parasite survival.^81^ *Leishmania donovani* deoxyhypusine synthase (DHS) was identified as an essential enzyme for parasite survival.^82^ This enzyme catalyzes the first step in the post-translational modification of eukaryotic initiation factor 5A (eIF5A). DHS34, specifically, plays a vital role in *L. donovani* survival, and due to its structural differences from human DHS, it presents a potential drug target. Given the activity of **62** and the essential role of DHS34, it is plausible that it binds to this target, providing a direction for further investigation.

**Figure 20:**
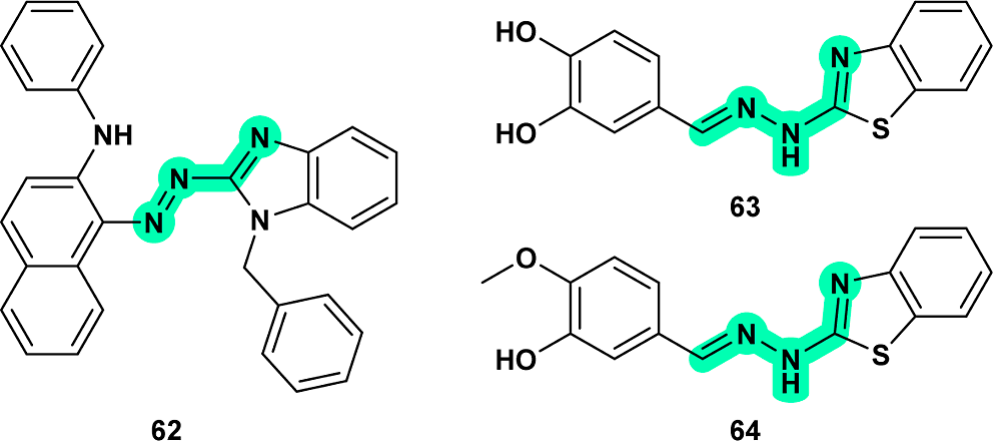
**MMV666023 (62**); **63** and its related compound **K060-0067** (**64**).

Benzothiazole derivative **63** (Figure 20) demonstrated significant efficacy against both the promastigote and intracellular amastigote forms of *Leishmania amazonensis*, surpassing the reference drug miltefosine in performance.^83^ Further investigation into its mechanism of action revealed that **63** induced mitochondrial dysfunctions, leading to depolarization of the mitochondrial membrane potential without triggering ROS production. Additionally, the analog of **63**, **K060**-**0067** (**64**), was included in a chemogenomic screening of 188 synthetic compounds, aimed at identifying bioactive substances.^84^ Although **64** exhibited extensive bioactivities and appeared to interact with specific genetic pathways in yeast models, particularly through gene deletion assays that mapped sensitivities and resistances, its exact molecular targets and mechanism of action were not fully defined, highlighting the need for further studies.

## 4 Overview of Relevant Targets Reported for Parent Compound and Close Analogues

The following table lists the compounds with a hydrazone scaffold and their analogues that have a known target and its respective *Leishmania* species for which each compound has already been tested. The relative change in potency, expressed as Δ­(logIC_50_), is also provided to highlight the variation in activity across different species or analogues (Table 1).

**Table 1:** Summary of Anti-leishmanial Activity, Relative Potency, and Putative Targets for the Screened Compounds

Compound ID	Leishmania species	relative Δ(logIC_50_)^a^	putative target	ref.
1	L. chagasi	–0.22	dihydrofolate reductase-thymidylate synthase	^17^
2	L. chagasi	+0.87	Leishmanial PERK	^17^
6	L. braziliensis	–0.58*	unknown	^26^
	L. amazonensis	+0.78*		
7	L. chagasi	+0.30	dihydrofolate reductase	^17^
9	L. donovani	+0.97^§^	DNA gyrase	^28^
11	L. donovani	–0.95	topoisomerase IIα	^31^
14	L. amazonensis	–0.52^§^	tyrosyl-DNA phosphodiesterase 1	^33^
16	L. panamensis	–1.20^§^	cysteine protease	^35^
18	L. amazonensis	+0.84	methionine sulfoxide reductase A	^37^
20	L. amazonensis	+0.05	interleukin-1 receptor associated kinase 4	^39^
	L. braziliensis	+0.09		
	L. peruviana	–0.05		
23	L. infantum	+0.21	DNA topoisomerases	^41^
24	L. panamensis	–1.48	unknown	^42^
25	L. infantum	undetermined	unknown	^43^
26	L. braziliensis	+2.18^+^	iron chelation	^44^
28	L. infantum	–0.09^+^	cysteine protease	^47^
30	L. amazonensis	–1.07	pyruvate kinase	^50^
32	L. infantum	–0.07	unknown	^51^
33	L. donovani	–1.73	KCNQ2 potassium channels	^52^
34	L. amazonensis	–0.00	unknown	^54^
37	L. infantum	undetermined	nitroreductases	^55^
39	L. amazonensis	–0.21*	ankyrin repeat protein	^57^
	L. braziliensis	+0.02*		
41	Leishmania donovani, L. major and L. enriettii	undetermined	leishmanial reductase	^58^
42	L. donovani	–0.88	unknown	^60^
44	L. donovani	–1.37	unknown	^60^
45	L. amazonensis	+0.09	cysteine protease	^64^
47	L. amazonensis	undetermined	METHIONINE AMINOPEPTIDASE 2	^66^
48	L. amazonensis	–2.42	METHIONINE AMINOPEPTIDASE 2	^67^
51	L. braziliensis	undetermined	unknown	^69^
53	L. braziliensis	+0.83^a^	unknown	^70^
54	L. infantum	undetermined	tyrosyl-DNA phosphodiesterase 1	^72^
56	L. donovani	–0.34*	unknown	^75^
58	L. infantum	–0.90	tyrosyl-DNA phosphodiesterase 1	^77^
59	L. infantum	undetermined	tyrosyl-DNA phosphodiesterase 1	^78^
60	L. major	+0.31	tyrosyl-DNA phosphodiesterase 1	^79^
62	L. major	undetermined	deoxyhypusine synthase	^80^
63	L. amazonensis	+0.25*	unknown	^83^

a Relative potency Δ­(−logIC_50_) = (−logIC_50_)_test_ – (−logIC_50_)_control_; *compared with Miltefosine, ^§^compared with AmB; ^+^compared with Glucantime.

Compounds **14** ^31^ (Figure 7) and **20** ^37^ (Figure 8), **54** ^71^ (Figure 17), **58**,^77^ **59**,^78^ and **60** ^79^ (Figure 19) are closely related compounds known to inhibit human TDP1. In *Leishmania donovani*, TDP1 plays a crucial role in repairing DNA damage caused by topoisomerase I inhibitors, such as **camptothecin**, by removing covalent topoisomerase I-DNA complexes, which would otherwise result in DNA fragmentation and cell death.^73^ Due to its key role in DNA repair, LdTDP1 is an attractive target for the development of new inhibitors designed to disrupt the DNA repair mechanism in *Leishmania*.^34,73^ This hypothesis may be explored for the design of potent, selective inhibitors targeting Leishmania TDP1.

Compounds **9** ^28^ (Figure 5); **18** ^37^ (Figure 8); **25** ^43^ (Figure 10); **34**,^54^ **39** ^57^ (Figure 13); **41**,^60^ **43** ^63^ (Figure 14); **47** ^66^ (Figure 15); **51** ^69^ (Figure 17); **56** ^75^ (Figure 18); **58**,^77^ **59** ^78^ (Figure 19), and **63** ^83^ (Figure 20) have been demonstrated by their respective authors to disrupt key mitochondrial features, potentially involving ROS-dependent mitochondrial signaling pathways, such as those mediated by methionine sulfoxide reductase A.^38^ Some of these disruptions may be linked to DNA biochemical processes, particularly through the inhibition of targets such as topoisomerase activity^32^ DNA gyrase,^39^ ankyrin repeat protein (XP_003723231.1) or TDP1 inhibition.^73^ Validating these assumptions may lead to the discovery of new potent mitochondrial disruptor compounds.

Cysteine proteases play a pivotal role in both *Leishmania* and *T. cruzi*, making them valuable targets for drug discovery despite structural differences between the cathepsin-like proteases in *Leishmania* and cruzain in *T. cruzi*. Compound **45** ^64^ (Figure 14) is active against *Leishmania* and closely resembles **46** ^65^ (Figure 14), which has demonstrated significant activity against *T. cruzi* through cruzain inhibition. Selenium-containing compounds, such as **28** ^47^ (Figure 11), have shown antileishmanial potential, while selenosemicarbazones (like **29** ^49^) (Figure 11) are recognized for their antichagasic activity as cruzain inhibitors. Related thioflavanone **16** ^35^ (Figure 7) is thought to bind to cathepsin L in *Leishmania*. Although binding between these proteases across species may not be directly relatable, it may aid in designing inhibitors with cross-species properties, warranting further exploration and validation.

## 5 Future Directions

Despite significant advances in understanding the antileishmanial potential of hydrazone-containing scaffolds, critical aspects of their mechanisms of action remain underexplored. Elucidating molecular targets, such as those involved in mitochondrial disruption and ROS-mediated signaling, is essential. Techniques like chemoproteomics, genetic screens, and metabolomics can unravel these mechanisms and identify pathways that are vital for parasite survival. This knowledge will support the rational design of derivatives with enhanced selectivity, efficacy, and reduced cytotoxicity.

The optimization of structure–activity relationships (SAR) is another priority for advancing hydrazone-based therapeutics. Computational approaches, including molecular docking and machine learning, combined with experimental SAR studies, can highlight pharmacophores critical for activity. These strategies will facilitate the design of second-generation compounds with improved bioavailability and pharmacokinetic properties, expediting their progression to preclinical development.

Synergistic therapies offer an exciting avenue for enhancing treatment efficacy and combating resistance. Combining hydrazone-based compounds with established antileishmanial agents, such as miltefosine or amphotericin B, can exploit complementary mechanisms of action. These combinations may also reduce toxicity and treatment duration, providing a robust approach to managing drug resistance while improving patient outcomes.

Lastly, specific molecular targets like TDP1, mitochondrial regulators, and cysteine proteases in Leishmania deserve further exploration. Investigating compounds that inhibit these targets can lead to dual-action therapies that simultaneously disrupt critical mitochondrial functions and DNA repair mechanisms. Such strategies hold promise for developing potent, selective, and affordable treatments, addressing the urgent need for effective therapies in endemic regions.

## Supplementary Material



## Notes

Molecular structures of all compounds in SDF format (ZIP)
